# Long-Term Outcomes of Restorelle® Direct Fix Anterior Mesh in the Treatment of Pelvic Organ Prolapse

**DOI:** 10.7759/cureus.63513

**Published:** 2024-06-30

**Authors:** Yi Man Goh, Shu Hui Lim, Hong Liang Chua, How Chuan Han, Jill C Lee

**Affiliations:** 1 Department of Urogynaecology, KK Women's and Children's Hospital, Singapore, SGP; 2 Department of Obstetrics and Gynaecology, KK Women's and Children's Hospital, Singapore, SGP; 3 Department of Urogynaecology, KK Women’s and Children’s Hospital, Singapore, SGP; 4 Department of Urogynaecology, HC Han Clinic for Women, Mount Elizabeth Novena Specialist Center, Singapore, SGP

**Keywords:** urinary incontinence, vaginal mesh complications, pelvic floor, pelvic organ prolapse, vaginal mesh

## Abstract

Objective

The objective of this study was to evaluate the efficacy and long-term outcomes of the use of Restorelle® Direct Fix (Coloplast, Humlebæk, Denmark) anterior mesh for transvaginal surgical management of anterior compartment prolapse.

Methods

A retrospective case series review was conducted for 123 patients who underwent surgery for Baden-Walker Grade three and four anterior compartment prolapse with the Restorelle Direct Fix anterior mesh between July 1, 2017 and September 30, 2018 in a single center. Follow-up was conducted at one, six, 12, 24, and 36 months after treatment. A standardized questionnaire and pelvic examination were conducted at each visit to assess operative complications and subjective and objective cure rates.

Results

Sixty patients were included in the analysis with a three-year follow-up rate of 70.0%. At three years post-operatively, subjective and objective cure rates were 97.7% and 95.3% respectively. Seven (11.7%) patients complained of de novo stress urinary incontinence, four (6.7%) complained of de novo urge urinary incontinence and one (1.7%) complained of symptomatic recurrence. Significantly, six (10.0%) patients had transvaginal mesh exposure over the three-year follow-up, mostly presenting within the first year. One (2.4%) patient developed new asymptomatic mesh erosion at the 36-month visit and one patient required mesh loosening one month post-surgery.

Conclusions

Management of anterior compartment prolapse with transvaginal surgery using the Restorelle® Direct Fix anterior mesh was associated with good subjective and objective cure rates. However, significant rates of post-operative mesh exposure were noted within three years post-surgery, which hinders the recommendation of this device for augmentation of repair for anterior compartment prolapse.

## Introduction

The incidence of pelvic organ prolapse requiring surgery ranges from 1.5 to 1.8 per 1000 women [1] and has the potential to significantly affect a woman’s quality of life. Treatment options include conservative management, but the mainstay of treatment for severe and symptomatic prolapse involves surgical repair. Since the introduction of transvaginal mesh surgery (TVM) in the 1990s, various studies comparing TVM and native tissue repair (NTR) have shown that TVM resulted in lower prolapse recurrence [2], anatomic failure [3] and repeat surgical repair compared to NTR [4]. However, high rates of stress urinary incontinence (SUI), mesh exposure, and bladder injury were reported [4-6] with the use of TVM. Due to the inadequate assessment of long-term safety of TVM devices and failure to demonstrate acceptable long-term benefits compared to NTR, the United States Food and Drug Administration (FDA) reclassified urogynecological mesh implants as class three (high risk) medical devices in 2016. Transvaginal mesh was ultimately withdrawn from the market in 2019 [7].

Restorelle® Direct Fix (Coloplast, Humlebæk, Denmark) anterior mesh is a four-arm, ultra-lightweight, pre-cut type one polypropylene transvaginal mesh used as reinforcement for transvaginal anterior compartment repair that has shown satisfactory technical feasibility. In this study, we aim to evaluate the efficacy and long-term outcomes of Restorelle® Direct Fix anterior mesh for surgical treatment of anterior compartment prolapse.

This article was previously presented as a meeting abstract at the 2023 Royal College of Obstetrics and Gynaecology World Congress, 12-24th June 2023.

## Materials and methods

A single-center retrospective observational study was conducted in KK Women’s and Children’s Hospital. Ethics approval was obtained from the local ethics board (CIRB reference number: 2018/2203). The electronic case notes of patients treated with Restorelle® Direct Fix anterior mesh-augmented repair between July 1, 2017 and September 30, 2018 were reviewed. Patients with Baden-Walker Grades three or four anterior compartment prolapse were included, regardless of other concomitant surgeries. The first 30 patients were excluded to account for the learning curve in performing the surgery, and the data for the remaining 93 patients were analyzed. 

Classification of the degree of pelvic organ prolapse by the Baden-Walker system was performed through clinical examination intraoperatively. Patients were assessed for associated urinary and bowel symptoms via a standardized clerking form. Pre-operative urodynamic testing was done routinely.

All surgeries were performed by the urogynecology team led by the same urogynecologist. In patients requiring concomitant hysterectomy, vaginal hysterectomy was done prior to TVM. Following this, vasopressin was infiltrated, and a midline incision was made over the anterior vaginal wall. The bladder was dissected from the anterior vaginal wall and the plane developed further to reach the sacrospinous ligament and the obturator membranes. The four-armed Restorelle® Direct Fix anterior mesh was introduced with its posterior arms fixed to sacrospinous ligaments and its anterior arms fixed to the obturator membranes using standard introducers provided in the mesh kit. Additional stitches were used to fix the mesh to the bladder fascia and anterior wall. The anterior vaginal wall was then closed with Vicryl Rapide 2-0. Posterior colporrhaphy was then performed for concurrent posterior compartmental and apical prolapse, and sacrospinous ligament fixation was done if indicated. Continence surgery was done after if required, and cystoscopy was performed to exclude bladder or ureter injury. After the surgery, the vagina was irrigated and packed with povidone-soaked gauze, and a transurethral Foley catheter was placed.

Operative details including the duration of surgery, estimated blood loss, and intra-operative and immediate post-operative complications were recorded. Patients were all admitted to the gynecological ward post-operatively and received standard post-operative care.

Follow-up intervals were arranged at one, six, 12, 24, 36, 48, and 60 months. A standard local questionnaire addressing urinary, pain, and recurrence symptoms was used (Appendix Table 6). Vaginal and speculum examinations were performed by attending physicians at each follow-up visit using a standardized form to evaluate the objective cure of prolapse and mesh complications. The objective cure was defined as post-operative prolapse of ≤ Grade one.

Data were collected and analyzed using IBM SPSS Statistics for Windows, Version 26 (Released 2019; IBM Corp., Armonk, New York, United States). Categorical data was shown using descriptive statistics; normally distributed data was shown as means (standard deviations) while non-normally distributed data was shown as medians (ranges). 

## Results

**Figure 1:**
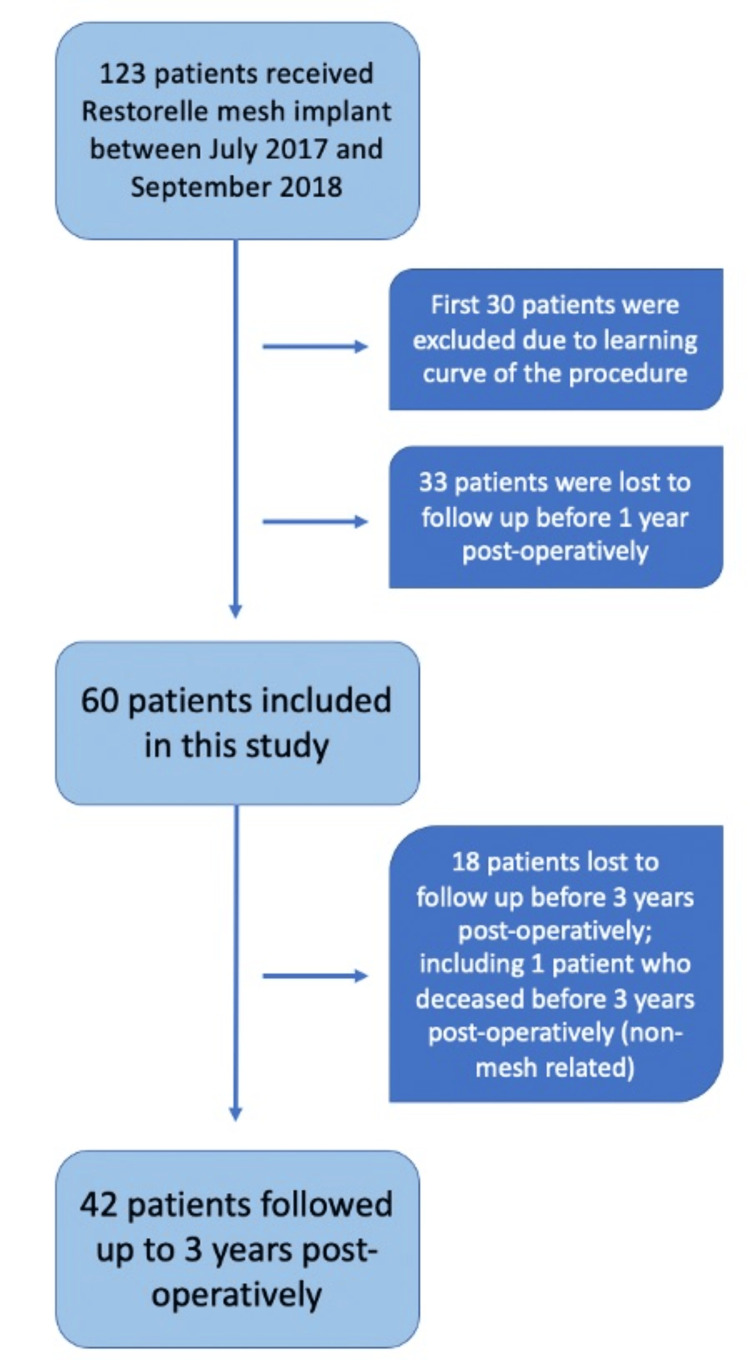
Recruitment and follow-up of the study population up to three years post-operatively

One hundred and twenty-three patients with Grades three and four anterior compartment prolapse had the Restorelle® Direct Fix anterior mesh kit inserted between July 1, 2017 and September 30, 2018. The first 30 patients were excluded due to the learning curve of performing the surgery. Thirty-three patients were lost to follow-up prior to their one-year post-operative follow-up, and 18 patients were lost to follow-up after three years including one patient who was deceased from a condition unrelated to the surgery (Figure 1). Sixty patients were followed up to the end of the first year, 49 to the end of the second year, and 42 to the end of the third year. A sample size of 60 was used to reflect demographics, perioperative findings, and immediate outcomes, 49 for symptoms and complications arising within the second year and 42 for those arising within the third year.

The demographic profile of the study population is summarized in Table 1. The mean age was 66.1 years, of whom 57 (95%) were menopausal and only 10 (16.7%) were sexually active.

**Table 1:** Profile and demographics of the study population *No patients had previous cesarean sections

Demographics	Mean ± SD (range)
Age (years)	66.1 ± 7.2 (50-81)
Body mass index (kg/m^2^)	25.1 ± 3.1 (18.6-32.6)
-	n (%)
Sexually active, n (%)	10 (16.7)
Parity (vaginal delivery) *	-
a) 0	1 (1.7)
b) 1	2 (3.3)
c) 2	22 (36.7)
d) 3	24 (40.0)
e) 4	7 (11.7)
f) >5	3 (5.0)
g) Data unavailable	1 (1.7)
Forceps/vacuum delivery	-
a) 0	57 (95.0)
b) 1	2 (3.3)
c) 2	1 (1.7)
Menopause	57 (95.0)
Hormone replacement therapy	1 (1.7)

Clinical assessment (Table 2) showed that all patients presented with sensation of lump at the introitus. Most had urinary symptoms such as urinary frequency (37, 61.7%), SUI (24, 40%), urge urinary incontinence (UUI) (27, 45%) and voiding difficulty (20, 33.3%). 38 (63.3%) patients had a Grade three anterior compartment prolapse while 22 (36.7%) had a Grade four anterior compartment prolapse. The majority of patients had concomitant posterior compartment prolapse. Of the 18 (30%) patients with previous gynecological surgery, five patients had previous pelvic floor repair, including one patient who had a previous TVM with a Prolift mesh.

**Table 2:** Pre-operative information and clinical examination SSF, sacrospinous ligament fixation; TVT-O, tension-free vaginal tape (obturator); PFR, pelvic floor repair; VH, vaginal hysterectomy; TAHBSO, total abdominal hysterectomy bilateral salpingoophorectomy

Preoperative Findings	n (%)
Urinary frequency/nocturia	37 (61.7)
Stress urinary incontinence	24 (40.0)
Urinary urgency	30 (50.0)
Urge urinary incontinence	27 (45.0)
Fecal incontinence	3 (5.0)
Lump at introitus	60 (100.0)
Voiding difficulty	20 (33.3)
Dyspareunia	5 (8.3)
Previous gynecological surgery	18 (30.0)
a) Total abdominal hysterectomy	6
b) VH/PFR/SSF/TVT-O	2
c) VH only	2
d) VH and posterior PFR	1
e) TVT-O/total PFR	1
f) TVT-O only	1
g) TAHBSO/PFR	1
h) Other gynaecologic surgeries	4
Demonstratable urinary stress test	4 (6.7)
Anterior compartment prolapse	-
a) Grade 3	38 (63.3)
b) Grade 4	22 (36.7)
Posterior compartment prolapse	-
a) None	1 (1.7)
b) Grade 1	6 (10.0)
c) Grade 2	32 (53.3)
d) Grade 3	10 (16.7)
e) Grade 4	7 (11.7)
Apical prolapse (Uterus intact), n (%)	48 (80.0)
a) Grade 1	0 (0)
b) Grade 2	4 (6.7)
c) Grade 3	1 (1.7)
d) Grade 4	43 (71.7)
Vault prolapse, n (%)	12 (20.0)
a) Grade 1	0
b) Grade 2	1 (1.7)
c) Grade 3	6 (10.0)
d) Grade 4	5 (8.3)

Intra-operatively, a large proportion of patients underwent concomitant surgery (Table 3). Forty-eight patients (80.0%) had vaginal hysterectomy with sacrospinous ligament fixation, 59 patients (98.3%) had posterior pelvic floor repair, while 25 (41.7%) had a mid-urethral sling. There were minimal intraoperative complications. Fever was the most common post-operative complication in 24 participants (40%) and majority of patients recovered after one day. Six patients had voiding difficulty, which led to prolonged catheterization after surgery ranging from 11 to 32 days. There were four (6.7%) patients who experienced buttock pain, and none had thigh pain. Two patients (3.3%) had vaginal vault hematoma, and one required surgical management. One of the two bladder injuries were directly related to placement of the mesh.

**Table 3:** Intra-operative details and perioperative outcomes *One case of bladder perforation from Restorelle mesh placement, one case from concurrent TVT-Exact #Rectal perforation during posterior repair

Intra-operative Findings and Perioperative Outcomes	
Anesthesia, n (%)	
a) General	42 (70.0)
b) Regional	18 (30.0)
Duration of surgery (min), mean ± SD (range)	119 ± 28 (56 – 200)
Concomitant surgery, n (%)	60 (100)
a) Vaginal hysterectomy	48 (80.0)
b) Anterior pelvic floor repair	6 (10.0)
c) Posterior pelvic floor repair	59 (98.3)
d) Sacrospinous ligament fixation	48 (80.0)
e) Mid-urethral sling	25 (41.7)
i. Altis	10 (16.7)
ii. Solyx	2 (3.3)
iii. TVT-Abbrevo	1 (1.7)
iv. TVT-Exact	12 (20.0)
f) Cystoscopy	60 (100.0)
Estimated blood loss (ml), mean ± SD (range)	259 ± 152 (50 – 800)
Intraoperative complications, n (%)	
a) Excessive blood loss >500ml	3 (5.0)
b) Blood transfusion	5 (8.3)
i. 1 unit	4 (6.7)
ii. 2 units	1 (1.7)
c) Bladder perforation *	2 (3.3)
d) Rectal perforation #	1 (1.7)
Hospital stay (day), mean ± SD (range)	5.2 ± 2.1 (2 – 15)
Catheterization (day), mean ± SD (range)	5.9 ± 7.1 (2 – 34)
Fever, n (%)	24 (40.0)
a) 1 day	11 (18.3)
b) 2 days	5 (8.3)
c) 3 days	5 (8.3)
d) >3 days	3 (5.0)
Postoperative complications, n (%)	18 (30.0)
a) Urinary tract infection	1 (1.7)
b) Buttock pain	4 (6.7)
c) Thigh pain	0 (0.0)
d) Voiding difficulty	6 (10.0)
e) Vaginal vault hematoma	2 (3.3)
f) Anemia	2 (3.3)
g) Other complications unrelated to mesh surgery	3 (5.0)

Sixty participants were followed up to one year, 49 up to two years and 42 up to three years. Subjective cure rates were determined by patient reported symptoms of a lump at introitus as well as patient satisfaction. There were high subjective cure rates of 100% three years post-operatively, with all 42 participants followed up to three years reporting no lump at introitus (Table 4).

**Table 4:** Three-year outcomes and comparisons (1 – 3 years) *One patient with recurrent rectocele also had recurrent cystocele # Mesh loosening one month post-op due to voiding dysfunction

Parameters	Onset of symptoms within			Total over three years (n=60)
	1 year post-op (n = 60)	2 years post-op (n = 49)	3 years post-op (n = 42)	
Abnormal urine flow, n (%)	1 (1.7)	2 (4.8)	0	3 (5.0)
De novo SUI, n (%)	2 (3.3)	3 (6.1)	2 (4.8)	7 (11.7)
De novo U/UI, n (%)	1 (1.7)	3 (6.1)	0	4 (6.7)
Wound dehiscence, n (%)	0	0	0	0
Mesh exposure	5 (8.3)	0	1 (2.4)	6 (10.0)
Sexually active, n (%)	5 (8.3)	9 (18.4)	9 (18.4)	-
a) Dyspareunia, n (%)	1 (1.7)	1 (2.0)	0	2 (3.3)
Lump	0	0	1 (2.4)	1 (1.7)
Pelvic pain, n (%)	1 (1.7)	1 (2.0)	0	2 (3.3)
Recurrent anterior compartment prolapse, n (%)	2 (3.3)	2 (4.8)	1 (2.4)	5 (8.3)
a) Grade 2	2	2	1	
Recurrent posterior compartment prolapse, n(%)	0	3 (6.1)	1 (2.4)	4 (6.7)
a) Grade 2	0	3*	1	
Recurrent vault prolapse, n (%)	0	0	0	0
Reoperation, n (%)	2 (3.3)	1 (2.0)	0	3 (5.0)
a) Mesh excision	0	1	0	
b) Mesh loosening	1#	0	0	
c) Mesh removal	1	0	0	
Satisfaction, n (%)	60 (100)	49 (100)	42 (100)	-
Subjective cure rate, n (%)	60 (100)	49 (100)	42 (100)	
Objective cure rate, n (%)	58 (96.7)	45 (91.8)	41 (97.7)	

There were also high objective cure rates of 97.7% post-operatively. Only five (8.3%) had recurrence of Grade two anterior compartment prolapses and four (6.7%) patients had Grade two posterior compartment prolapses three years post-operatively.

However, there was significant mesh exposure rates seen in patients over three years post-operatively; six (10%) patients had an onset of asymptomatic mesh exposure throughout the study period, 5 of which occurred within the first-year post-operatively. Of six patients with mesh exposure, there was resolution after application of vaginal estrogen cream, while one patient had persistent mesh exposure two and three years post-operatively requiring mesh trimming in clinic. One patient underwent mesh loosening of anterior aspect of the mesh at one month post-operatively due to voiding dysfunction. The details of mesh extrusion cases are summarized in Table 5.

**Table 5:** Details of mesh extrusion cases, symptoms, and treatment given

Age of the patient during surgery	Onset of mesh extrusion	Size and location	Dyspareunia	Pelvic pain	Intervention	Resolution
57	1 month	3mm interior 2mm vault	Not sexually active	Nil	Data unavailable	Resolved at six months
50	1 month	2mm mesh exposure at anterior 3cm below urethral meatus	Not sexually active	Nil	Premarin cream given	3mm x 3mm felt at six months post-op, resolved thereafter
77	12 months	1cm x 1cm Vaginal apex	Not sexually active	Nil	Premarin cream given	Unresolved
	24 months	1cm x 0.5cm felt	Not sexually active	Nil	Trimmed with scissors in clinics	Unresolved
	36 months	1cm x 0.5cm felt	Not sexually active	Nil	Trimmed with scissors in clinics	Data unavailable
70	12 months	Not documented	Not sexually active	Nil	Premarin cream given	Mild mesh felt at two years post-op
59	13 months	Not documented	Not sexually active	Nil	Data unavailable	Resolved at two years post-op
71	36 months	1cmx0.2cm mesh extrusion Anterior wall near vault	Not sexually active	Nil	Premarin cream given	Data unavailable

## Discussion

Transvaginal mesh augmented repair for anterior compartment prolapse remains controversial due to concerns regarding safety, with mesh exposure and chronic pain being the most feared complications. These concerns have contributed to the withdrawal of transvaginal mesh from the market in 2019 by the US Food and Drug Administration. Previous data has shown high cure rates and low recurrence of prolapse in mesh-augmented repair compared to native tissue repair [2,3]. Recurrence rates for native tissue repair are reported to be close to 40%, with the anterior compartment being the most common site of recurrence [8]. No differences were observed in the risk of re-operation between the two surgical approaches [9]. Chan et al. found that TVM is associated with the most favorable outcomes in women with advanced POP [10], suggesting that patient selection plays a crucial role in determining success of mesh placement.

The three-year subjective and objective cure rates in this study are high, similar to the ElevateTM anterior mesh used in the same institution, which reported a subjective cure rate of 100% and an objective cure rate of 96.2% five years post-operatively [11]. A Chinese study by Huang et al. reported similar cure rates of 94-99% at three years follow-up [12]. High subjective cure rates and satisfaction rates have been attributed to the small percentage of patients who remain sexually active, although all the sexually active patients in our study did not experience subjective recurrence of prolapse.

Small mesh exposures may be treated conservatively with topical vaginal estrogen, while larger exposures may be distressing for patients and require surgical management. Long-term outcomes for TVM have been published for similar devices [2,13,14] such as the ElevateTM Anterior mesh kit [11], but there is limited data on long-term outcomes on Restorelle [15]. Mesh exposure rates with the ElevateTM anterior mesh in our institution were reported to be up to 3.8% over five years. Gauthier et al reported a 2.9% rate of mesh exposure with Restorelle Direct Fix anterior mesh over 12 months, but no studies with long-term follow-up are available [16]. In comparison, the mesh exposure rates in our three-year study are higher (10%), but the majority of mesh exposures were subcentimetre in length and the patients did not have dyspareunia or pelvic pain. They were treated conservatively with vaginal estrogen, or trimmed with scissors in the outpatient clinics, with only one case requiring surgical excision of the mesh.

There are no studies explaining the higher mesh exposure rates of the Restorelle Direct Fix anterior mesh compared to Elevate anterior mesh and Gynecare Prolift. Liang et al. discussed that the loss of porosity of meshes after applying an axial force could decrease the biocompatibility of said mesh, resulting in bridging fibrosis, inflammation and poor tissue integration [17]. He postulated that the Restorelle mesh, being less likely to change shape causing reduction in porosity while axial force is applied, is a more stable mesh which could result in less fibrosis and inflammation. A separate study comparing different TVM applications in non-human primates over 12 weeks also showed that a less stiff Restorelle mesh was better incorporated into tissue compared to stiffer alternatives [18].

In transvaginal mesh surgery, there is risk of intraoperative injury to surrounding vascular structures, nerves, and organs such as the bladder and rectum [19]. These complications may range from mild to severe and have a significant impact on patient recovery and satisfaction. Fortunately, the rates of severe complications were low in this study. Three (5.0%) cases had excessive blood loss of more than 500ml, which is comparable to a study in Austria that reported increased intraoperative bleeding as the most common complication from transvaginal mesh surgeries (6.8%) [20]. Dissection in vascular areas such as the obturator space and around the sacrospinous ligaments during mesh insertion and concomitant transvaginal surgery is a likely contributing factor.

There were two (3.2%) cases of bladder perforations and one (1.6%) case of rectal perforation intraoperatively, of which only one (1.6%) case of bladder perforation was due to the application of mesh. A French study by Ferry et al included 218 patients who had concurrent pelvic floor repair with Restorelle Direct Fix anterior mesh that reported comparable complication rates of bladder perforations and rectal perforations (0.5% respectively) [15]. A Russian study in 2022 reported rectal injury of 1%, also occurring during the dissection of the vaginal wall such as in this study [21]. Overall, the immediate complication rate from surgery with transvaginal mesh is low.

In the longer term, complications associated with mesh surgery include de novo SUI and UUI, dyspareunia, and recurrent low-grade anterior and posterior compartment prolapse [19]. Two (3.3%) patients experienced de novo SUI within one year of follow-up, with an additional three (6.1%) and two (4.8%) diagnosed at two and three years respectively. The 6.7% de novo UUI rate can be postulated to be confounded by the concurrent mid-urethral sling surgeries performed at the time of transvaginal mesh placement. This is a known complication of mid-urethral sling placement [22]. Low rates of dyspareunia reported (3.3%) could be explained by the low rates of sexual activity in the patient population. Only five patients were sexually active within the first year post-operatively, and five more resumed sexual activity more than one year post-operatively. Notably, the sexually active patients did not experience any mesh exposure, although sexual activity has been known to increase the risk of mesh exposure [23]. Anterior and posterior compartment recurrence rates were low, with no patient experiencing symptoms of a lump at introitus, and no patient having objective severe prolapse (Grade three or four). Patient satisfaction rates remained high at 100%, as most patients only experienced transient symptoms which resolved with conservative management.

Study strengths included that patients were recruited under a single trained surgeon, which eliminates surgical abilities confounding outcomes. Patient factors and assessment of outcomes were standardized, as only patients with grade three to four anterior compartment prolapse received mesh augmented repair while outcomes were assessed with a standardized questionnaire throughout a three-year follow-up. 

The limitations of the study include its retrospective nature, which was associated with incomplete data, and can result in observer bias. This study is also a non-comparative study, as there is no comparison made to outcomes of patients undergoing native tissue repair. There is also a high drop-out rate beyond the 36-month period due to the pandemic, as such data up to 60 months was not collected as originally intended. Further prospective and comparative studies on Restorelle are unfortunately no longer possible due to withdrawal of the product by the U.S. Food and Drug Administration in 2019.

The other limitations are that all patients who received the Restorelle implant in this study had concomitant surgeries as illustrated in Table 3, which may confound complication rates. Interobserver differences during post-operative follow-up may result in small differences in assessment of recurrent prolapse but did not affect recognition of key complications such as mesh exposure.

## Conclusions

In the context of an aging population and longer life expectancy, pelvic organ prolapse is becoming a significant disease, causing serious effects on quality of life. Safe and effective methods of treatment are in demand, and the introduction of the transvaginal mesh was initially promising in view of the reported lower recurrence rates compared with traditional native tissue repair. The primary aim of this study was to evaluate the effectiveness and potential pitfalls associated with the use of Restorelle Direct Fix anterior mesh, one of the transvaginal meshes introduced into the market.

The findings of this study demonstrate the device’s efficacy, with high objective and subjective cure rates within three years of follow-up. Immediate complication rates were low. However, significant rates of mesh exposure, which can have a detrimental effect on quality of life, hinder the ability to recommend this device for the treatment of pelvic organ prolapse. Larger-scale comparative studies are needed to better determine the efficacy and safety of this device in the treatment of pelvic organ prolapse. Unfortunately, due to the withdrawal of the device from the market, these studies may not be completed.

## Appendices

**Table 6:** Standardized questionnaire for follow-up review SUI: Stress urinary incontinence; UUI: urge urinary incontinence

Standardized questionnaire for follow-up review	
Subjective symptoms	
Subjective parameter	Response
Abnormal urine flow	Yes/No
De novo SUI	Yes/No
De novo UUI	Yes/No
Dyspareunia	Yes/No/NA
Lump felt	Yes/No
Pelvic pain	Yes/No
Patient satisfaction after surgery	Yes/No
* If the patient answered yes to any of the above symptoms, please let them elaborate on how severe their symptoms are and how they have affected you. Please also allow them to elaborate on how satisfied they are with the surgery.	
Objective assessment	
Mesh erosion	Yes/No
Recurrent anterior vaginal wall prolapse	Grade 0/1/2/3/4
Recurrent posterior vaginal wall prolapse	Grade 0/1/2/3/4
Recurrent vault prolapse	Grade 0/1/2/3/4
Reoperation	Yes/No
* Please indicate the size and site of mesh erosion, if any. Please elaborate on the indication and type of reoperation, if any.	
